# Syndrome of Inappropriate Antidiuretic Hormone Secretion (SIADH) and Subsequent Central Diabetes Insipidus: A Rare Presentation of Pituitary Apoplexy

**DOI:** 10.1155/2019/7592648

**Published:** 2019-04-02

**Authors:** S. B. Smedegaard, J. O. Jørgensen, N. Rittig

**Affiliations:** ^1^Department of Internal Medicine, Horsens Regional Hospital, Sundvej 30, 8700 Horsens, Denmark; ^2^Department of Diabetes and Hormone Diseases (DoH), Aarhus University Hospital, Palle Juul-Jensens Blvd 99, 8200 Aarhus N, Denmark; ^3^Steno Diabetes Center Aarhus, Aarhus University Hospital, Palle Juul-Jensens Blvd 99, 8200 Aarhus N, Denmark

## Abstract

Pituitary apoplexy (PA) is a rare endocrine emergency that occasionally presents with sodium disturbances. Here we present a rare case with a previously healthy 41-year-old female who presented with acute onset headache and nausea without visual impairment or overt pituitary dysfunction. Plasma sodium concentrations declined abruptly during the first two days of admission to a nadir of 111 mmol/l. Urine and blood chemistry were consistent with syndrome of inappropriate antidiuretic hormone secretion (SIADH). Magnetic resonance imaging revealed recent bleeding into a pituitary cystic process. Hyponatremia was successfully corrected with fluid restriction and both visual function and anterior pituitary function remained intact. Subsequently, the patient developed central diabetes insipidus (CDI), which responded well to desmopressin substitution. To our knowledge, this is the first case of PA presenting predominantly with posterior pituitary dysfunction that transitioned from SIADH to permanent CDI.

## 1. Introduction

Pituitary apoplexy (PA) is a rare clinical emergency induced by sudden haemorrhage or infarction into the pituitary gland, which predominantly occurs into an existing pituitary adenoma [1]. Abrupt onset of severe headache is the main symptom, but neurological symptoms, nausea, visual impairment, ocular palsy, and endocrine disturbances frequently occur [1–3]. Severe hyponatremia due to the syndrome of inappropriate antidiuretic hormone secretion (SIADH) is occasionally reported in PA [4–7] and also occurs in ≈ 6% of patients undergoing pituitary surgery [8].

In general, SIADH is caused by inappropriate excretion of vasopressin from the pituitary gland, ectopic vasopressin production, or increased ADH sensitivity [9]. The condition was first detected by William Schwartz and Frederic Bartter in 1967, who also established the clinical criteria for SIADH, which includes serum sodium < 135 mmol/l, serum osmolality < 275 mmol/kg, urine sodium > 40 mmol/l, urine osmolality > 100 mmol/kg, absence of clinical signs of volume depletion, absence of known conditions causing hyponatremia, and successful correction of sodium levels with fluid restriction [10]. Various conditions can cause SIADH including granulomatous diseases, malignant tumours, central nervous disease, and drug intake [9]. Rare causes of SIADH such as PA may be overlooked, not least since the two conditions share symptoms and signs including nausea and hyponatremia.

Central diabetes insipidus (CDI) is another rare condition, which is characterized by polyuria (> 3 L per day), excessive thirst, polydipsia, and elevated serum sodium levels [11]. It is known that CDI complicates PA with a prevalence of less than 5% and that the condition most often is temporary and resolves within the following weeks [1, 3]. To our knowledge, a case of PA dominated by SIADH and subsequent CDI in the absence of overt anterior pituitary deficits has not previously been reported.

## 2. Case Presentation

**Figure 1:**
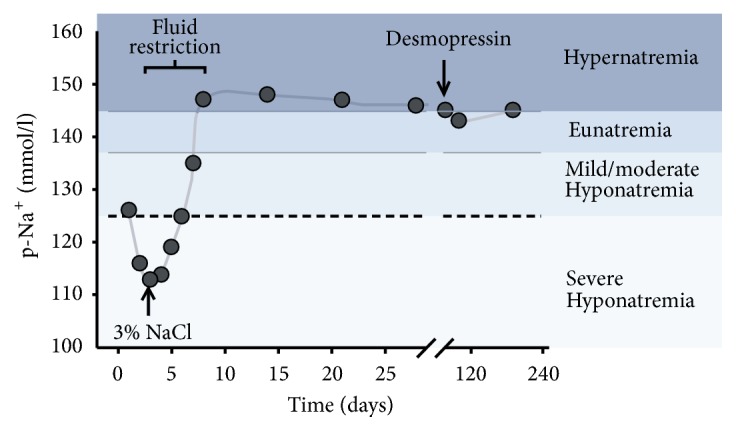
Plasma concentrations of sodium. Horizontal axis depicts time course from hospital admission (day 0) until 180 days' follow-up. Vertical axis depicts plasma sodium concentrations (p-Na^+^). Hyponatremia was treated with intravenous hyperosmolar sodium infusion (3% NaCl at day 2) and fluid restriction (day 2 to day 5). The subsequent development of central diabetes insipidus (CDI) was treated with low dose desmopressin (initiated around day 60).

**Figure 2:**
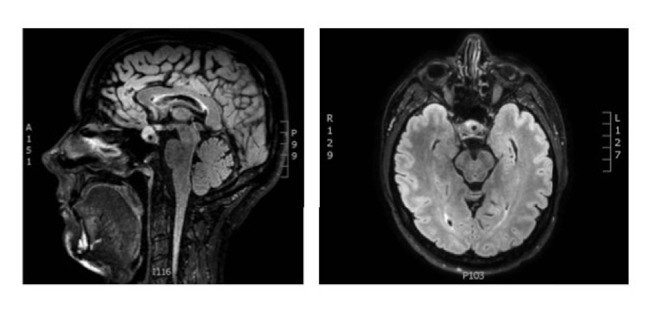
Magnetic resonance imaging of cerebrum. Magnetic resonance imaging (MRI) showed signs of recent bleeding into a cystic adenoma comprising 10x10x8 mm placed in sella turcica with close proximity to the chiasma and displacing the pituitary gland to the right.

A previously healthy 41-year-old Caucasian woman was admitted to the Emergency Department at the Regional Hospital of Horsens with a three-day history of severe headache, nausea, and dizziness. The physical examination was unremarkable with no evidence of impaired vision. Regular medication only included oral contraceptives (75 microgram desogestrel). Initial blood screen tests revealed moderate hyponatremia (126 mmol/l) and borderline low levels of iodothyronines (T_3_ and T_4_) and thyroid-stimulating hormone (TSH, Table 1). An acute cerebral computed tomography (CT) did not show haemorrhage or infarction, and no mass lesion in the sellar region. Lumbar puncture showed no signs of infection or bleeding. Additional blood tests showed normal anterior pituitary function (Table 1) except moderate hyperprolactinemia. During the first two days of admission, plasma sodium concentrations dropped to a nadir level of 111 mmol/l (Figure 1). On the third day, a magnetic resonance imaging [MRI] of the brain showed recent bleeding into a cystic process (10x10x8 mm) in the sellar region in close proximity to the optic chiasm with displacement of the pituitary gland to the right (Figure 2). Urine and blood examination at day two (Table 1) were consistent with SIADH according to standard criteria [10]. The patient was treated with fluid restriction (day two to day five) and an intravenous bolus of hypertonic saline 3% (day two only), which induced a gradual increase in plasma sodium concentrations (Figure 1). During the following weeks, the patient developed polyuria, polydipsia, and persistent hypernatremia. She was diagnosed with central diabetes insipidus (CDI) and successfully treated with desmopressin (dose 0.1 mg daily).

A MRI follow-up after three and ten months showed no change in the size of the cystic adenoma and automated perimetry showed a normal visual field. Anterior pituitary function remained intact, whereas the patient's CDI is considered permanent.

## 3. Discussion

Pituitary apoplexy is a rare but acute condition causing severe headache often associated with visual disturbances including ocular palsy, and altered consciousness. The clinical picture resembles that of subarachnoid haemorrhage (SAH) or meningitis, which often delays the diagnosis [1]. Pituitary imaging confirms the diagnosis by revealing a haemorrhagic or necrotic pituitary tumour [12]. Corticotropic deficiency causing adrenal insufficiency must be evaluated and promptly treated, and acute surgery is indicated if the symptoms worsen [1]. The present case of PA is unique owing to its presentation with isolated posterior pituitary dysfunction that transitioned from SIADH into permanent CDI.

It is well known that neurosurgery and brain trauma may cause hyponatremia, which is often attributable to SIADH [13–15]. Furthermore, it is evident that pituitary surgery may elicit a tri-phasic response with acute hypernatremia, transient hyponatremia around postoperative day 7 (between 3 and 11 days), and subsequent development of CDI [16–19]. Our case shows a similar course with SIADH/hyponatremia approximately 5 days following initial symptoms and development of CDI in the ensuing weeks. We therefore speculate that PA may cause sodium disturbances through similar mechanisms as pituitary surgery and brain trauma, which include an acute outburst of ADH from damaged cells in the posterior pituitary gland [14, 15]. This damage may cause subsequent CDI that typically resolves within weeks but occasionally result in permanent CDI [14].

Hyponatremia is the most frequent electrolyte disturbance, especially in hospitalized patients [20], and PA is a rare and often overlooked cause of hyponatremia. Most emergency departments use standard blood test screens at admission, and cerebral CT-scans are also widely used in this setting. Studies have shown that CT only detects 21% of PA, whereas 90% is visualized with MRI [12, 21]. The absence of visual impairment and lack of clear anterior pituitary affection undoubtedly delayed the MRI and hence the final diagnosis in our case.

Pituitary apoplexy remains an important albeit rare differential diagnosis in patients presenting with severe and acutely onset headache, and our case illustrates that PA may masquerade as isolated SIADH and develop into CDI.

## Figures and Tables

**Table 1a:** (a) Standard blood test screen at admission

*Na* ^*+*^	126	↓	*(137 - 145)*	*mmol/l*
*K* ^*+*^	3.5		*(3.5 - 4.6)*	*mmol/l*
*CRP*	1.8		*(<8)*	*mg/l*
*Leucocytes*	6.4		*(3.5 - 10)*	*10* ^*9*^ */l*
*TSH*	0.4		*(0.3 - 4.5) *	*10* ^*3*^ * IU/l*
*T*_*3*_	3.1	↓	*(3.9 - 6.8)*	*pmol/l*
*T*_*4*_	11.4	↓	*(12 - 21)*	*pmol/l*

**Table 1b:** (b) Extended blood and urine tests

*Prolactin*	1375	↑	*(90 - 580)*	*10* ^*3*^ * IU/l*
*ACTH*	36		*(7 - 64) *	*ng/l*
*Cortisol 0 min*	541			*nmol/l*
*Cortisol 30 min*	797		*(> 500)*	*nmol/l*
*FSH*	23			*IU/l*
*LH*	6.7			*IU/l*
*Estrogen*	<15			*pmol/l*
*IGF-1*	192		*(70-210)*	*μg/l*
*Osmolality*	241	↓	*(280-300)*	*mmol/kg*

Urine				
*U-Osmolality*	744		*(300 - 900)*	*mmol/kg*
*U-Na* ^*+*^	140			*mmol/l*

Biochemistry: standard blood test screen and extended blood and urine tests. This table shows selected blood sample concentrations from the standard blood test screening performed on the day of hospital admission and the extended blood test analysis performed on day two of admission. Parenthesis illustrates the normal range. ↓ = below normal range. ↑ = above normal range. *CRP= C reactive protein, TSH = thyroid stimulating hormone, T*_*3*_*= triiodothyronine, T*_*4*_*= thyronine, ACTH= adrenocorticotropic hormone, FSH= follicle-stimulating hormone, LH= luteinizing hormone, and IGF-1= insulin-like growth factor 1.*

## Abbreviations

CDI: Central diabetes insipidus
CT: Computed tomography
MRI: Magnetic resonance imaging
PA: Pituitary apoplexy
SAH: Subarachnoid haemorrhage
SIADH: Syndrome of inappropriate antidiuretic hormone secretion.
